# 

**Published:** 1983-10

**Authors:** 


Contents

161. Editorial
162. Some aspects of Medical Research in the U.K.
Brandon Lush
165. Malignant Melanoma Incidence and association with Arsenic
R. Philipp, A. O. Hughes, M. C. Robertson and T. F. Mitchell
170. Triple Synchronous Colo-Rectal Carcinoma causing Intestinal Obstruction
Arthur Allan
173. A Survey of Double Contrast Barium Enemas at Bristol Royal Infirmary
P. G. P. Stoddart and J. Virgee
176. Imaging Techniques in the Diagnosis of a Mediastinal Mass
J. Bell, J. F. O'Reilly, G. Laszlo, P. Wilde and Paul Goddard
181. From our Correspondents
184. From our Foreign Correspondent
185. South West Orthopaedic Club
188. West Country Physicians Club
190. South West Radiologists Association
198. The Surgical Club of South West England
201. Book Reviews

*continued overleaf*

Established 1883

### BRISTOL MEDICO-CHIRURGICAL JOURNAL

Incorporating the Medical Journal of the South West

202. **Correspondence**
203. **Programmes of Societies**
205. **Examination Results**

